# Research and Application of Knowledge Resources Network for Product Innovation

**DOI:** 10.1155/2015/495309

**Published:** 2015-03-25

**Authors:** Chuan Li, Wen-qiang Li, Yan Li, Hui-zhen Na, Qian Shi

**Affiliations:** School of Manufacturing Science & Engineering, Sichuan University, Chengdu 610065, China

## Abstract

In order to enhance the capabilities of knowledge service in product innovation design service platform, a method of acquiring knowledge resources supporting for product innovation from the Internet and providing knowledge active push is proposed. Through knowledge modeling for product innovation based on ontology, the integrated architecture of knowledge resources network is put forward. The technology for the acquisition of network knowledge resources based on focused crawler and web services is studied. Knowledge active push is provided for users by user behavior analysis and knowledge evaluation in order to improve users' enthusiasm for participation in platform. Finally, an application example is illustrated to prove the effectiveness of the method.

## 1. Introduction

In the era of knowledge economy and network economy, enterprises need product innovation imminently in the face of fierce market competition. The open innovation strategy as an emerging approach towards innovation is receiving attention from more and more institutions and researchers [1]. Open innovation mode not only requires taking advantage of internal knowledge resources for innovation, but also emphasizes the importance of external knowledge resources for enterprise innovation [2]. Modern product design is based on knowledge, and knowledge acquisition is the core of the product design process [3]. Knowledge supporting product design process of ten covers multiple industries, disciplines or fields. Thus, Internet has become the main way to acquire, transmit, and share the knowledge. With the development of web information technology, various types of knowledge resources for product design keep on increasing explosively and massive information resources that distribute disorderly scatter in every corner of the Internet.

At the same time, researchers in the field of product design assistance models and systems apply to modeling development in conceptual design. These studies include ontology-based models for TRIZ [4–6], RFBS model of conceptual design [7], and ontological function design knowledge model [8]. These modeling techniques have different ontologies and building modules. However, these building modules are not always available in conceptual design.

In conceptual design of products requiring design knowledge across engineering domains and disciplines, it is essential for designers and engineering disciplines to communicate, share knowledge and information, and collaborate [9]. Many design systems have been developed, which include the following: Zhu and Xie propose establishing the universal description, discovery, and integration knowledge resources registration model [10]. Li et al. build a new framework model of the knowledge service platform for modern design with the integration design and flow planning theory and methods [11]. Cao et al. carry out the key technologies and methods of design knowledge acquisition from the network resource in the distributed resources environment and present the framework of the network resource integration platform [12]. He et al. present the share strategies and construction methods of distributed conceptual design knowledge resource, which are centered on design task and based on dynamic alliance without agency [13]. Cai et al. put forward a new intelligent discovery method for manufacturing resource based on semantic web, and a prototype system SWMRD is presented to provide manufacturing resources services in the Internet [14]. Hu et al. propose an industry oriented network knowledge discovery and sharing service platform to fast and accurately obtain the knowledge from industry network resources [15].

Network resources have many characteristics, including complex types, diverse forms, huge numbers, and low structuring. The existing research provides some ideas to establish integration platform and sharing network resources but neglects the importance of users who are the audience of knowledge resources service. Users more passively accept knowledge resources provided by the platform, lacking of initiative and participation. When a user is faced with a large number of knowledge resources, if the platform can offer an active knowledge push in accordance with users' need, to a certain extent, users' burden about the utilization of platform will be reduced. At present, domestic and foreign research on knowledge push more concentrates in the push system [16, 17], recommendation algorithm [18, 19], knowledge push rules [20, 21], including the research in terms of the product design work flow [22, 23], but few researches are focused on knowledge push for product innovation. This paper focuses on the way of acquiring knowledge resources supporting product innovation design from the Internet and providing active knowledge push, to reduce the blindness of users' knowledge resources searching in the design process.

Based on existing research, a method of implementation is put forward in this paper. A network knowledge resources integration framework is built, through ontology modeling for knowledge oriented innovation in Section 2. The technology of network knowledge resources acquisition is studied in Section 3. By using a focus crawler, knowledge resources of general sites are acquired, and professional database resources are described combining with web services. Considering the importance of users, active knowledge push based on users' behavior is provided to enhance users' viscosity of the platform in Section 4. The methodology of product innovation platform is applied to a case study as described in Section 5. Finally, the last section gives conclusions and suggestions for future work.

## 2. Integration Framework of Knowledge Resources Network

Knowledge demand of different stages in product innovation design process is highly different. The concrete implementation of the whole process is under the support of a variety of design resources with multidisciplinary and multifield knowledge interacting with each other, which enables designers to jump out of the limitation of existing design experience, to achieve a higher level of product innovation. Knowledge is part of innovative design. It is refined and summarized from the past research and facts, which has a positive role in guiding for further creative activities. Innovative design needs us not only to make use of knowledge, but also to go beyond the limits of existing knowledge [24], through unified modeling of knowledge resources, to provide knowledge service for users.

### 2.1. Knowledge Model Based on Ontology

**Figure 1:**
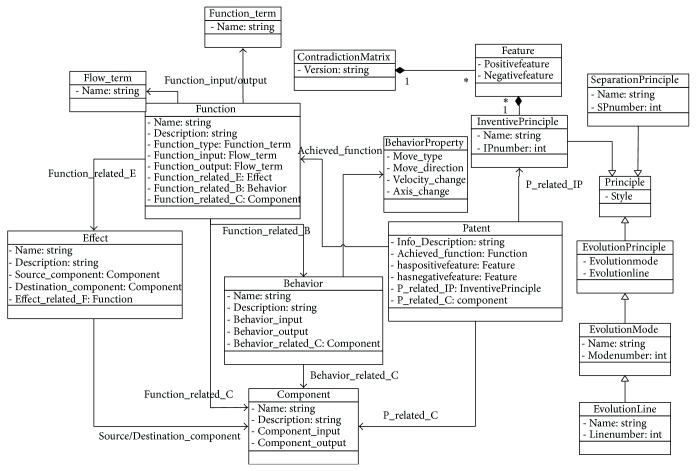
The basic structure of knowledge model based on ontology.

In this paper, ontology is used to standardize knowledge support for innovative design so as to facilitate the sharing and reuse of design knowledge. In ontology layer of integration framework, ontology can be formally described as a 5-tuple: KO = (C, P, I, R, A). C is a set of concepts. P is a set of properties related to concepts, which is the description of characteristics of concept. I is a set of concept instances, formed by instancing concepts. R is a set of relations, used to represent the interaction relationship between concepts, such as the relationships between global and local represented by part_of, inheritance relationships represented by kind_of, the relationships represented by attribute_of that a concept is a property of another concept, and the relationship between the instances represented by instance_of. A represents the axioms and rules, to provide some correlations and constraints for concepts, properties, and relationships; for example, parts or components consist of one or more parts. The basic structure of knowledge model based on ontology is shown in Figure 1.

We choose functional basis in the form of “verb + noun” to represent function. Verbs describe function type and nouns describe flow. Function_term and Flow_term, respectively, represent the set of function terms and the set of flow terms. Function_type represents the type of functions, provided by Function_term. Function_input and Function_output, respectively, represent input flow and output flow of functions, provided by Flow_term. Function_related_B represents the behavior of function implementation, which expresses the mapping from function layer to behavior layer. Function_related_C represents the structure of function implementation, which expresses the mapping from behavior layer to structure layer. In F-B-S model, behavior is a bridge connected function with structure. Description of behavior includes the movement behavior and motion mechanism of behavior implementation. Behavior_related_C represents relational structure of movement behavior implementation. Behavior_input and Behavior_output, respectively, represent the input and output characteristics of movement behavior, which are described by BehaviorProperty, including Move_type, Move_direction, Velocity_change, and Axis_change. Each property has a specific value; for example, Move_type has a value with moving, rotating, and synthesis of movement. Structure is the carrier of concrete implementation from function to behavior. Component_input and Component_output, respectively, represent main body to implement input and output behavior.

Scientific effect through some principles converts input to output for the implementation of relevant functions. Effect class is related to Function class by Effect_related_F. Source_component and Destination_component, respectively, represent source component and target component, corresponding to the input and output flow from effect to function. In patent class, Info_Description contains the basic information such as name, applicant, time, and number. haspositivefeature and hasnegativefeature, respectively, represent improved and worsened parameters related to the patent, which are related to Structure and Function by P_related_IP and P_related_C. Principle contains invention principle, separation principle, and evolutionary theory. There is a relationship between separation principle and invention principle. Conflict matrix consists of 39 engineering parameters and 40 invention principles. Each two engineering parameters (improved and worsened parameters) make up an engineering parameter pair. Each engineering parameter pair has relations with invention principle, and each invention principle may correspond to some subprinciples. Evolutionary theory contains different technical evolution modes, and a technical evolution model is composed of several technical evolution routes.

### 2.2. Knowledge Resources Network Integration Framework

**Figure 2:**
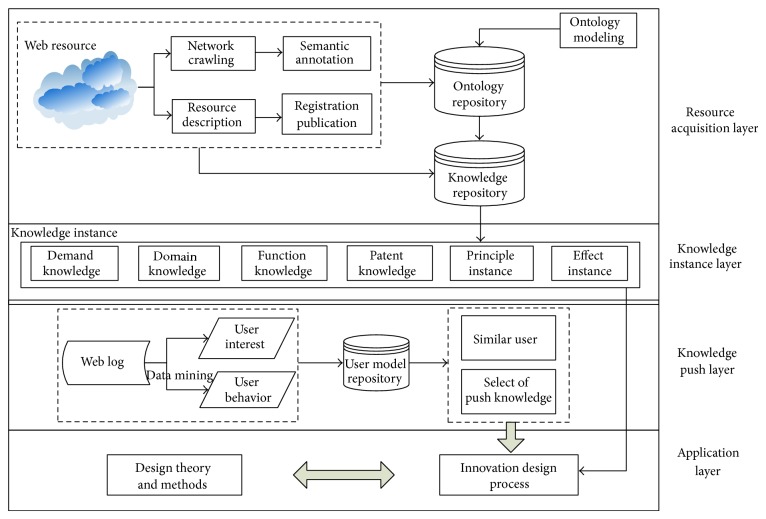
Knowledge resources network integration framework.

Knowledge resources network integration framework of product innovation design platform is shown in Figure 2, mainly divided into four layers. Through the integration framework, a large amount of knowledge of the related design resource is acquired in the network and the relationship between kinds of knowledge is established, to provide active knowledge push and support designers for innovative design.

*(1) Resource Acquisition Layer*. According to knowledge ontology modeling, the relationship between kinds of knowledge is established and organized. A consistent representation of knowledge resources is provided for semantic description. Ontology provides a formal representation of relationships and concepts which belong to design knowledge oriented innovation, in order to achieve abstract expression of knowledge on the semantic level. Through ontology semantic classification and labeling, a series of ordered and systematic annotation instances are obtained from knowledge resources, in the way of the network crawling and the registration of web services. Annotation instances obtained from the network are stored in the repository under the organization of ontology.

*(2) Knowledge Instance Layer*. On one hand, the static knowledge instances analyzed by researchers are stored in the platform. On the other hand, the dynamic knowledge instances acquired in the way of the network crawling and the registration of web services are also stored. The static or dynamic knowledge instances can be seen as further expansion of the concept and relationship of the knowledge ontology. There is a mutual link relation between knowledge instances under the effect of ontology organization. Besides, knowledge instances provide intellectual support for design activities combining innovation design theory and utilizing innovation design process in the application layer.

*(3) Knowledge Push Layer*. User model repository contains all kinds of user model, and we had explained the construction of a specific user model in detail in [25]. With the mining of web log, the actual condition of user behaviors, such as scoring, collecting, sharing, commenting, browsing, and clicking, and users' interest about knowledge are gained. Finally, according to finding the similar users, relevant knowledge is pushed in the system.

*(4) The Application Layer*. In this layer the related services of product innovation design are provided, including product innovation design process and product innovation design theory support for design process. Innovation design theory is applied to every stage of product innovation design process. For example, in the requirements analysis phase, common methods we used include cluster analysis, analytic hierarchy process, and quality function deployment (QFD). These methods can be divided into creative thinking method based on psychology, innovation design method based on knowledge, the research of invention problem based on engineering technology development rule, and innovation method based on artificial intelligence.

### 2.3. The Application Model of Knowledge Resources Network

**Figure 3:**
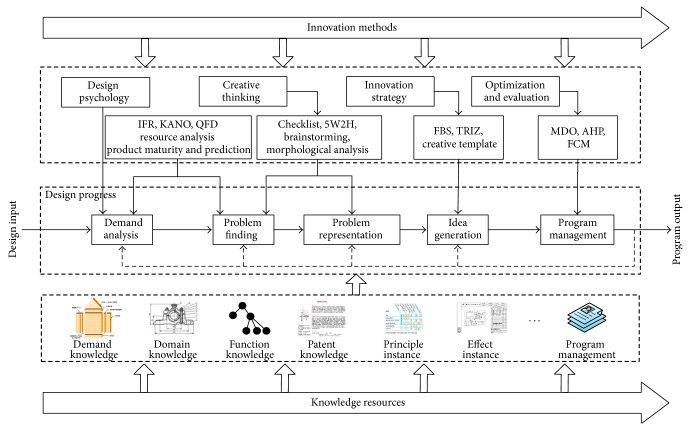
The application model of knowledge resources network.

In the innovation design process, internal and external knowledge is integrated for each design process. At the same time, combined with “person,” the main design body, knowledge resources are constantly enriched and improved in designer's using process. The application model of knowledge resources network is shown in Figure 3.

A designer starts a design task from demand analysis generally. In the process of demand analysis to problem finding, knowledge instance such as demand knowledge, patent knowledge and principle instance are provided for the design process. The analysis tools such as IFR, KANO, and QFD provide support for the problem finding process methodologically. Problem representation is critical in the design process. At this stage, based on design information and their own experience and relevant knowledge, the designer analyzes problems through creative thinking such as 5W2H and brainstorming. After problem representation, different innovation strategies are used to solve problems to complete idea generation. At the stage of idea generation, one or more creative idea may be produced. Combining product performance, constraints, and other demands, one or more original solutions are chosen to fit the specific application conditions, in the comparison with lots of instances. Program management is to optimize or evaluate the design results. For example, we can use AHP or FCM to evaluate the solution with the goal of performance or economy.

## 3. The Acquisition of Knowledge Resources Online

Product innovation design platform has a need to integrate many kinds of knowledge resources online. Its main goal is to gain available design resources in the environment of vast amounts of knowledge resources and finally to guide and aid designers to innovate. In domestic and overseas there are many related websites including industry websites, providing information of products for insiders, such as http://www.jx.cn/, http://www.made-in-China.com/, and http://www.mechnet.com.cn/; data resource websites, which are professional databases or connected to database, such as escience, 5ipatent, and wanfangdata; software portal websites, providing software information based on some innovation method, such as creax and iwint; knowledge service websites, namely, providing knowledge services, such as baike.baidu, zhidao.baidu, and ishare.sina. From the perspective of product design, these sites can be seen as knowledge resources support for product innovation design.

**Figure 4:**
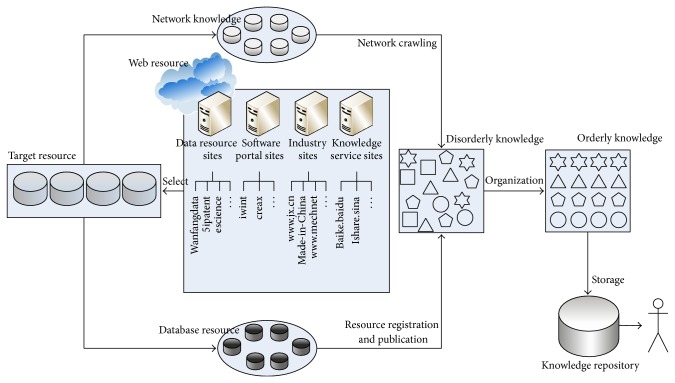
The acquisition process of knowledge resources online.

The acquisition process of knowledge resources online is shown in Figure 4. Because the network information is multiple and heterogeneous, we must make a choice, picking out websites related with design from the Internet sites. Obtaining and collecting useful knowledge from the target resources can be in two ways: regularly using the network crawling or describing, publishing, and calling resources via the web service. After obtained knowledge is organized, knowledge would be stored in repository. Finally, orderly knowledge will be pushed for users.

### 3.1. Focused Crawler for Web Knowledge Resources

**Figure 5:**
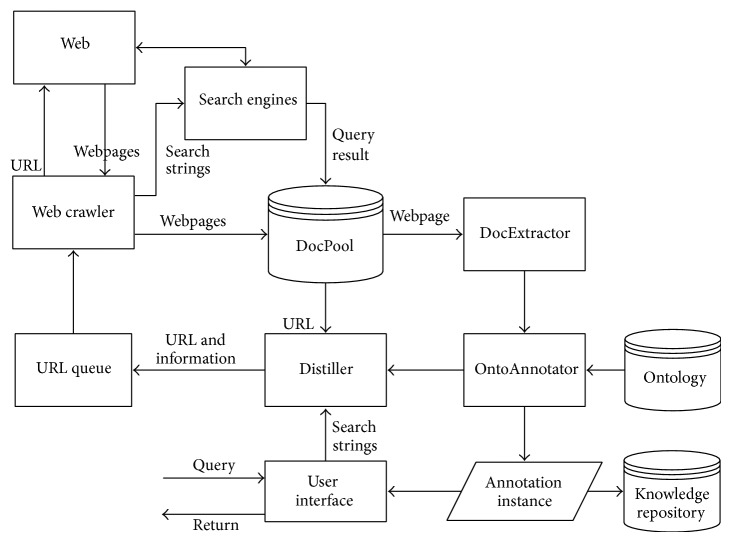
The network focused crawler process.

Knowledge resources are automatically collected by the network crawling, which is the mainstream means to obtain web information resources at present. General web crawler is only focused on the acquisition of URL link of webpages and grabbing of pages or pages block overall, but the basic content of the pages will not be processed. Different from general web crawler, focused crawler does not pursue a large coverage but aims to grab pages related to a particular topic, so as to prepare data resources for topic-oriented users' query [26]. In order to provide fast, accurate, and stable results of search engine in the semantic level, a focused crawler method based on ontology is proposed [27]. Based on the method above, the network focused crawler process is shown in Figure 5 in this paper, including the following.

1. The directional grabbing of the webpage. According to the target URL, web crawler downloads and grabs webpage. User's query is retrieved on the web through search engines such as Baidu and Google. Based on search results above, the relevant pages will be grabbed by web crawler. Then the webpages retrieved from the web are uniformly stored in DocPool, for analyzing and processing of webpage in the next step.
2. Webpage document analysis and ontology-based classification of captured pages. When analyzing the webpage in DocPool, firstly we use DocExtractor to obtain some basic webpage information: HTML Analyzer analyzes the HTML structure of webpage to get URL, title texts, and heading texts. HTML TAG Filter eliminates tags from the webpage. Webpage without tags is sent to OntoAnnotator for processing: use ontology to annotate features with term frequencies for each class, and learn to crawl the characteristics and classify the webpage.
3. Storing the semantic annotation instance to the repository, providing users for search. The annotated webpages in which term frequency reaches the threshold value are stored to the repository. Distiller decides the importance of webpage, giving a URL queue to determine the turn of page crawling dynamically.

### 3.2. The Acquisition of Database Resources

For the purpose of security or other reasons, some database resources only allow having a specific visit on its own platform. Databases are mostly heterogeneous, so we need to use web service technology to acquire database resources. Description of online resources, interview method, and discovery method between different providers is defined by web service. Because web service with an integrated ability completely shields the difference between software platforms, it makes the distributional resources online to form a virtual computer system. An important problem involved the reuse of resources is the description of resources [28].

Database resource description is the following triples, DBResourse = (U, DBInfo, DBMetadata). U represents the URL for the database resources. DBInfo is the basic information of the database resources. DBInfo = (ID, Name, Description, Source, Updatetime, Connection); details are shown in Table 1. The most essential definition of metadata is “data about data” or “data about the containers of data.” Knowledge resource metadata is “data about the knowledge resources,” the so-called “knowledge of knowledge” [29]. DBMetadata mainly includes the description of content and structure; details are shown in Table 2. Functional basis, invention principle, technical parameters, and other specific properties of design knowledge can be represented as a field.

The description of data resources should be interpreted easily by computer. XML (Extensible Markup Language) with flexible structural has become the standard for data representation and exchange and provides a free format syntax, which is the source language that allows the user to define his own markup language. According to the establishment of an XML document, web service can be described, structured description of data resources based on XML as shown in Algorithm 1.

## 4. Knowledge Active Push

### 4.1. Knowledge Push Based on User Behavior

**Figure 6:**
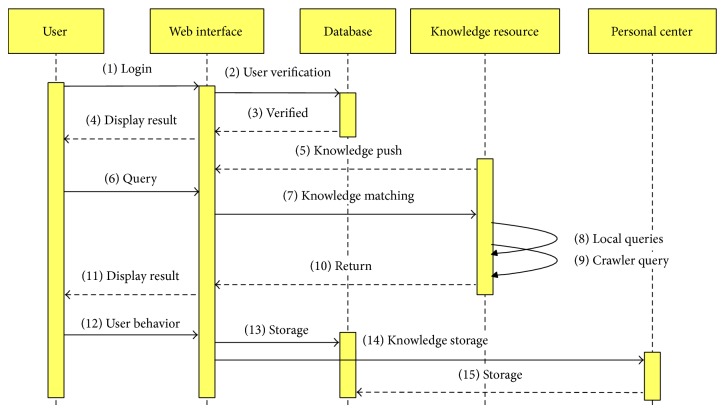
Knowledge push based on user behavior.

User behavior can reflect users' interest to some extent. Different people have different user behavior. When knowledge resources are used, a wide variety of user behaviors will be generated. According to the form of behavior, user behavior is divided into explicit and implicit behavior [30]. Explicit feedback of user behavior refers to these behaviors that can reflect the need of user for knowledge directly, such as scoring, collecting, and sharing. Implicit feedback of user behavior cannot obviously decide the need of user, such as comment, browse, and click. In web logs, there are a lot of user behaviors. Based on statistical analysis of user behavior like collecting, sharing, commenting, browsing, and clicking and others, you can obtain users' demand for knowledge resources.  Figure 6 is the UML sequence diagram of knowledge push based on user behavior.

When the platform is used for product innovation design, we need to view or search for related knowledge resources to get incentives. These knowledge resources can be derived from the instances inside of the platform and also come from knowledge annotation instances based on focused crawler and web services. While the searched knowledge resources return to web interface, users will produce many behaviors such as grading, collecting, sharing, commenting, browsing, and clicking in terms of the actual use of knowledge in the design process. Then such user behaviors are saved in the platform. When a user visits a piece of knowledge and decides to save it in his personal center, it is indicated that this piece of knowledge can meet the demand of the user to a large extent. Next time the user logs in, the platform will find similar users based on user behavior in the system and push out the relevant knowledge.

### 4.2. Select of Push Knowledge

Knowledge as an essential asset plays an important role in innovation and gradually formed in the design activities [31]. Knowledge manipulation activities operate on knowledge resources to create value for an organization. The value generation depends on the availability and quality of the knowledge resources [32]. Knowledge resources evaluation appears in the process of knowledge browsing. Users' feedback of knowledge resource evaluation can reflect the value of knowledge. Knowledge value can be evaluated from the five essential aspects, which are detailed in the following.

Explicitness indicates the explicit extent of knowledge, whether it is easy for users to understand. Novelty implies the extent of knowledge whether to be new or to be old. Importance indicates how significant the knowledge is for a user in the process of knowledge utilization. Availability signifies the potential value of knowledge from the perspective of user. Correlation is used to reflect the extent that the knowledge provided is relative to the user. Evaluation of the value of knowledge is shown in Table 3.

Based on score from the user, the evaluation vector for a piece of knowledge is indicated by *R* = (*K*_1_, *K*_2_, *K*_3_, *K*_4_, *K*_5_). Wherein *K*_1_, *K*_2_, *K*_3_, *K*_4_, and *K*_5_, respectively, stand for explicitness, novelty, importance, availability, and correlation, *K*_1_, *K*_2_, *K*_3_, *K*_4_, *K*_5_ ∈ {1,2, 3,4, 5}. Similarity between users implies the extent of similarity between the various vectors. We choose the modified cosine similarity method to calculate the similarity between target user and other users, computation formula as follows:

(1) $$\begin{array}{c} \text{sim}\left(i,j\right)=\frac{\sum_{c\in I_{ij}}\left(R_{ic}-{\overset{-}{R}}_{c}\right)\left(R_{jc}-{\overset{-}{R}}_{c}\right)}{\sqrt{\sum_{c\in I_{ij}}{\left(R_{ic}-{\overset{-}{R}}_{c}\right)}^{2}\sum_{c\in I_{ij}}{\left(R_{jc}-{\overset{-}{R}}_{c}\right)}^{2}}}. \end{array}$$

*I*_*ij*_ stands for the evaluation item sets which are graded by the user *i* and *j* commonly. *R*_*ic*_ represents the score of evaluation indicator *c* graded by user *i*. ${\overset{-}{R}}_{c}$ indicates the average score of evaluation indicator *c*. As the number of knowledge instances in knowledge resources network is relatively large and the number of similar users via the similarity calculation above may not be small, the calculation is more time-consuming to push knowledge concerned by all similar users. Therefore, we only select the top-*N* value to push knowledge, and the specific value of *N* can be set based on the real environment.

When a target user enters into the platform, the system will provide some knowledge that may be useful for target user according to searching for similar users. Then we select the appropriate knowledge from that concerned by similar users for the target user. When user *n* has scanned knowledge *i* and decides to store it to personal center, user *n* will give a score *P*(*n*, *i*) which signifies the extent of demand for *i*. Sim(*n*, *m*) indicates the similarity between user *n* and *m*. Similarity is dynamic; the system needs to update it timely. *N*_*m*_ is a set of similar users. The potential demand value for user *n* with knowledge *i* is calculated as follows:

(2) $$\begin{array}{c} \text{score}\left(m,i\right)=\frac{\sum_{n\in N_{m}}\left(P\left(n,i\right)*\text{sim}\left(n,m\right)\right)}{\sum_{n\in N_{m}}\text{sim}\left(n,m\right)}. \end{array}$$

We can set a threshold value; if score(*m*, *i*) > *a*, the knowledge is pushed for the user.

## 5. A Case Study

Based on the integrated framework and implementation methods above, our research group has discussed the technology of knowledge resources acquisition and knowledge active push in product innovation platform. The platform employs ASP.NET technology with Visual Studio as the integrated development environment and Microsoft SQL Server as the database management system for data storage.

**Figure 7:**
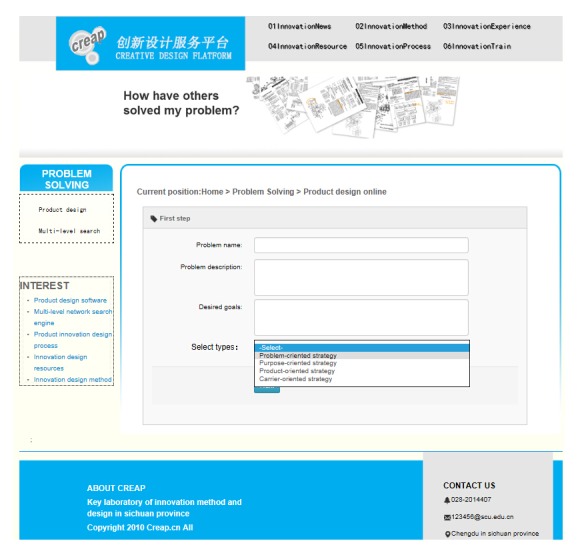
Interface of innovation design.

**Figure 8:**
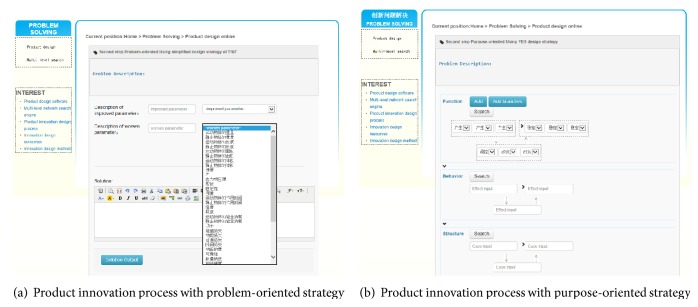
Interface of product innovation process with different strategies.

Figure 7 shows the interface where the user enters the module of innovation design. First, the client's requirements are inputted into the system. The problem name is defined by the user, and it is ultimately saved as the name of output solution. The problem description needs the user to describe the problem briefly so that designer can organize his thoughts and make the problem needed to be addressed clearly. Desired goals help users to make the goal clear and definite in product design process, to prepare for the choice of design type in the next step. Second, we choose appropriate strategy for product conceptual design. With the platform in the form of dropdown list for users to choose design types, the user can choose different strategies (the problem-oriented strategy, purpose-oriented strategy, product-oriented strategy, and carrier-oriented strategy) for product design. And the product conceptual process can be browsed via the graphical interface as shown in Figure 8.

The design type of problem-oriented strategy is based on TRIZ method. According to the problem description, we complete the description of improved and worsened parameters. Then we choose the improved and worsened parameters in dropdown list. The platform will provide invention principle related to the parameters. Finally, we can find relevant knowledge in the repository with the invention principle. The design type of purpose-oriented strategy is based on FBS method. We complete the product design process step by step with mapping from function to behavior and to structure. First, by the way of adding nodes, we determine the function schema of product, by selecting appropriate functions described with natural language in the form of dropdown list. Second, the functional basis is used as keyword to search for relevant effect from local effect repository and network. Then clients use effects to search for relevant structure from local structure instance repository and other web resources. By comparing with different structures, we choose appropriate structure to develop the structure framework. Finally, we choose the effective solution to save in our personal centers.

**Figure 9:**
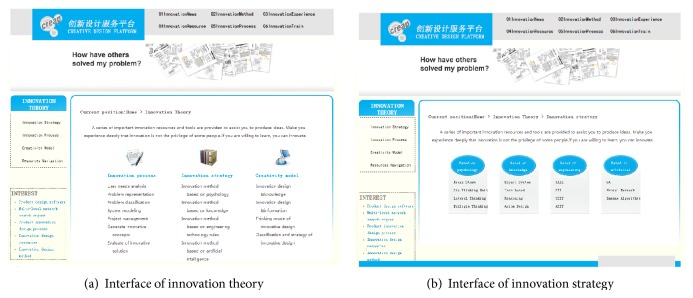
Interface of innovation method.

Figure 9 illustrates the graphical interface for innovation method of product innovation design. Graphical and literature knowledge are provided to a user for browsing different innovation methods, by which users can get more enlightenment and inspiration in product innovation design process.

**Figure 10:**
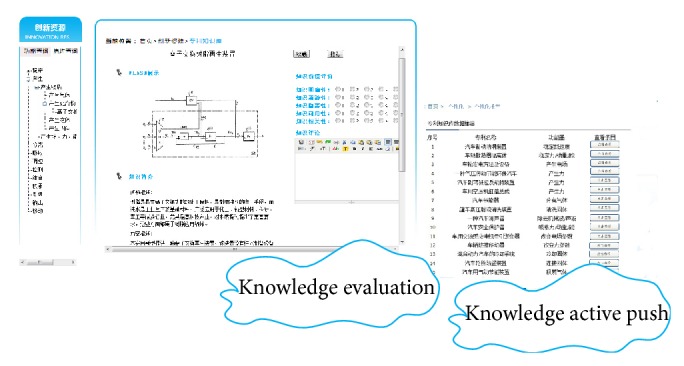
Interface of knowledge evaluation and active push.

Figure 10 illustrates the graphical interface for knowledge evaluation and knowledge active push. Graphical and literature knowledge from patent repository are shown in this figure. After a designer reads this piece of knowledge, he may evaluate this knowledge through some behaviors, like grading, collecting, sharing, and commenting. When the knowledge push button is clicked, pieces of relative knowledge are pushed to the designer.

## 6. Conclusion

Considering the importance of users, this paper proposes a method of acquiring knowledge resources supporting product innovation from the Internet and providing knowledge active push, to enhance knowledge service capacity of the platform and reduce blindness of users' knowledge resources searching in the design process. This method can be used to obtain knowledge resources supporting product innovation design from the Internet and improve the knowledge serving capability. Through the acquisition of users' behavior and the analysis of users' interest, the platform can provide knowledge active push to help designers to innovate.

Though significant progresses have been made on product innovation platform, there is still a lot of work to be done in the future, such as the development of ontology knowledge model to improve the construction, the development of product innovation platform to improve the search speed, and the optimization of knowledge push algorithm in the platform.

## Figures and Tables

**Algorithm 1:**
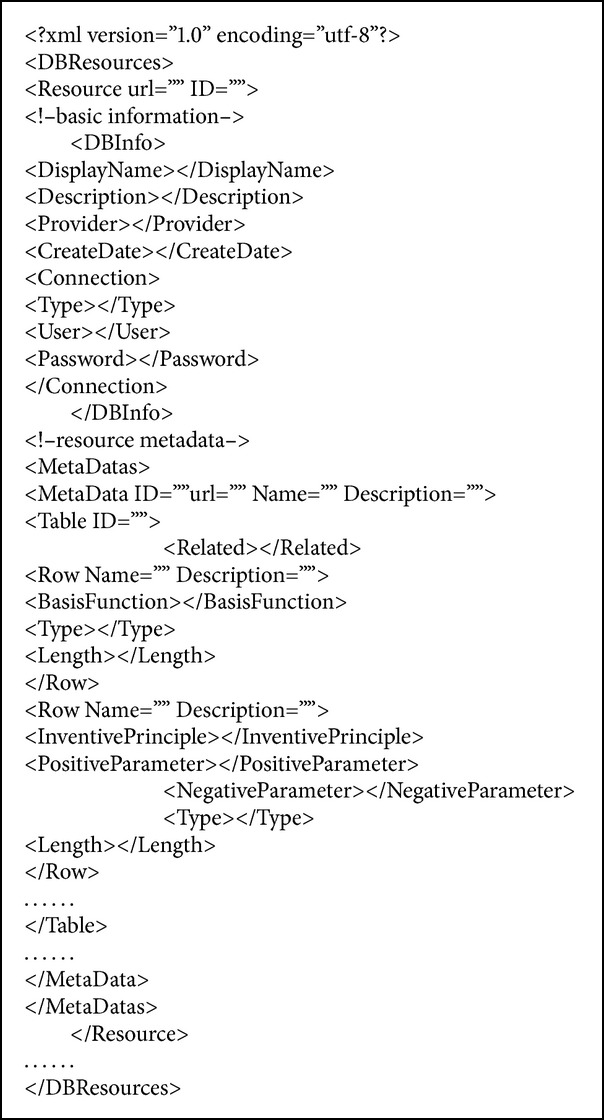


**Table 1:** Basic information of database resources.

Attribute	Explanation
ID	Unique name of data resources must exist and not be empty
Name	Resource name displayed to the user cannot be empty
Description	The explanation of data resources' content, scope, and application
Source	Unit or organization offering data resources
Updatetime	Update time that data resources upload
Connection	Login verification provided to connect data resources cannot be empty

**Table 2:** Information of metadata.

Attribute	Explanation
Form ID	Form ID of data resource must exist and not be empty
Name	Form name displayed to the user cannot be empty
Description	Description about content and application of the data within the form
Field	Definition of a field including name, type, and length
Metadata	Provide links to other metadata

**Table 3:** Evaluation of the value of knowledge.

Score	Explicitness	Novelty	Importance	Availability	Correlation
1	Not clear	Obsolete, ordinary	Insignificant	Unworkable	Irrelevant
2	Partly understood	Partly novel	Partly important	Partly available	Weakly related
3	Elementarily clear	Innovative enough	General important	Have certain effect	Generally related
4	Reasonable content	Comparative novel	Relatively important	Highly available	More relevant
5	Clearly definite	Unprecedented	Very important	Very applicable	Very relevant
